# 2-Styrylchromones: Cytotoxicity and Modulation of Human Neutrophils’ Oxidative Burst

**DOI:** 10.3390/ph15030288

**Published:** 2022-02-25

**Authors:** Mariana Lucas, Marisa Freitas, Marco Zanchetta, Artur M. S. Silva, Eduarda Fernandes, Daniela Ribeiro

**Affiliations:** 1 LAQV, REQUIMTE, Laboratory of Applied Chemistry, Department of Chemical Sciences, Faculty of Pharmacy, University of Porto, 4050-313 Porto, Portugal; mflucas@ff.up.pt (M.L.); marisafreitas@ff.up.pt (M.F.); 2 LAQV, REQUIMTE & Department of Chemistry, Campus de Santiago, University of Aveiro, 3810-193 Aveiro, Portugal; m.zanchetta3@campus.unimib.it (M.Z.); artur.silva@ua.pt (A.M.S.S.); 3 Faculty of Agrarian Sciences and Environment, University of the Azores, 9700-042 Angra do Heroísmo, Portugal

**Keywords:** styrylchromones, reactive oxygen species, reactive nitrogen species, inflammation, structure-activity relationship

## Abstract

Neutrophils are polymorphonuclear leukocytes recruited to sites of acute inflammation, in response to pathogen invasion and tissue injury. The modulation of their activity, especially oxidative burst, may be important to control the inflammatory process. 2-Styrylchromones (2-SC) are derived from chromones and despite their recognized multiple biological activities, their anti-inflammatory and antioxidant properties are still poorly explored. Therefore, in this study, 43 structurally related 2-SC were evaluated concerning their effects on freshly isolated human neutrophils’ viability and oxidative burst. The studied 2-SC were divided into eight groups according to their substitution at C-4′ on B-ring (none, -OH, -OCH_3_, -OBn, -CH_3_, and -NO_2_), existence and location of -Cl on B-ring, and presence of -Br at C-3 on C-ring. Overall, most of the studied 2-SC did not affect neutrophils’ viability, at physiological relevant concentrations. The ones belonging to B group were the most effective (IC_50_ values < 2 μM), and present one -OH group at C-4′ or a catechol group at C-3′ and C-4′ on B-ring. These substituents seem to play an important role in the modulatory activity of human neutrophils’ oxidative burst. These results reinforce the great potential of 2-SC’s scaffold for the development of new anti-inflammatory agents.

## 1. Introduction

**Figure 1:**
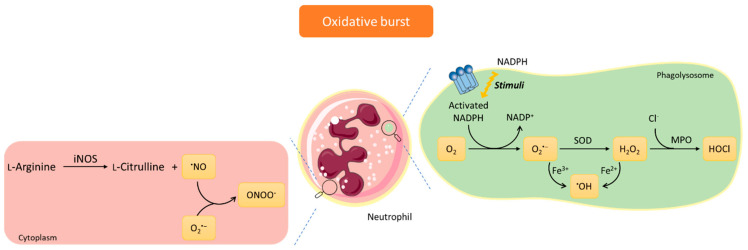
Mechanisms involved in production of reactive species in neutrophils’ oxidative burst. The nicotinamide adenine dinucleotide phosphate (NADPH) oxidase complex is activated, its cytoplasmic components migrate to the cellular membrane, and the molecular oxygen is reduced to the superoxide anion radical (O_2_^•−^), which in turn is the precursor to hydrogen peroxide (H_2_O_2_) and other reactive species [3,4,7,8]. Most of the H_2_O_2_ formed is consumed by myeloperoxidase (MPO), which catalyzes the formation of hypochlorous acid (HOCl), in the presence of H_2_O_2_ and by oxidation of the halide Cl^−^, since this ion exists in high concentrations in the body [4,7,8,9]. In the cytoplasm, L-arginine can be converted into L-citrulline and nitric oxide radical (^•^NO) by the action of the inducible nitric oxide synthase (iNOS). Consequently, ^•^NO can react with O_2_^•^^−^ and form peroxynitrite anion (ONOO^−^) [8,9,10]. ^•^OH, hydroxyl radical; SOD, superoxide dismutase.

Inflammation is involved in the development and worsening of several diseases, such as diabetes, rheumatoid arthritis, and cancer [1]. Inflammation is a natural biological process by which the body triggers a non-specific protective response due to harmful stimuli, namely pathogen invasion and tissue injury [1,2]. At the onset of inflammation, cellular signaling occurs, prompting the release of a series of inflammatory mediators. These mediators will increase the permeability of endothelial cells enabling immune cells, such as neutrophils, to access the site of infection or injury [1,2]. Neutrophils belong to the polymorphonuclear leukocytes group, together with basophils and eosinophils, and are highly specialized white blood cells characterized by their rapid migration to the inflammatory local and by their important role in the defense of the organism [3]. At the inflammatory site, neutrophils are activated and trigger a cascade of mechanisms to eliminate the invading agents. One of the main mechanisms by which neutrophils eliminate the invading agents is through the activation of nicotinamide adenine dinucleotide phosphate (NADPH) oxidase complex, with the consequent production of reactive species [4,5]. The reactive species produced during oxidative burst (Figure 1) are essential for the effective antimicrobial and anti-inflammatory activity of neutrophils [3,4]. However, the overproduction of these reactive species, characteristic of the chronic inflammatory diseases, can compromise the endogenous antioxidant defenses, leading to an imbalance between the amount of reactive species produced and their removal, resulting in a phenomenon known as oxidative stress. Consequently, this condition can cause harm to the host, particularly near inflamed tissues, namely through the oxidative damage of biomolecules, such as DNA, lipids, and proteins [2,6].

**Figure 2:**
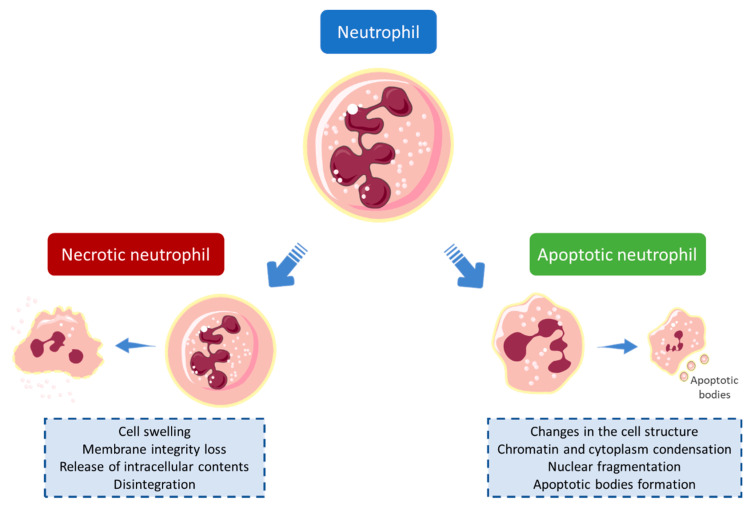
Mechanisms involved in cell death (apoptotic and necrotic pathways). During necrosis, the cell swells, which consequently leads to the loss of membrane integrity and the release of intracellular contents, and subsequently the disintegration of the cell. The apoptotic process is characterized by condensation of chromatin and cytoplasm, fragmentation of the cell nucleus, and formation of apoptotic bodies. In addition, changes in cell structure occur [13,15,16].

Neutrophils are immune cells with a short life span, which homeostasis is essentially maintained by constitutive apoptosis upon aging. This type of cell death is a programmed process characterized by several specific cellular changes manifested in the nucleus, cytoplasm, mitochondria, and plasma membrane (Figure 2) [11,12]. Another of the main death mechanisms is necrosis, which is characterized by certain hallmarks as cell swelling, plasma membrane rupture, and release of intracellular content (Figure 2) [13]. During the inflammatory process, there is a delay in apoptosis, which significantly increases the longevity of neutrophils, ensuring their continued presence at the site of inflammation [5,14]. Although these cells are fundamental in the inflammatory process, their activity and survivability must be controlled, to avoid an extended exacerbation of inflammation. Therefore, the development of strategies capable of modulating neutrophils’ activity, namely oxidative burst, has become a central point in the search for anti-inflammatory therapeutic agents [14].

**Figure 3:**
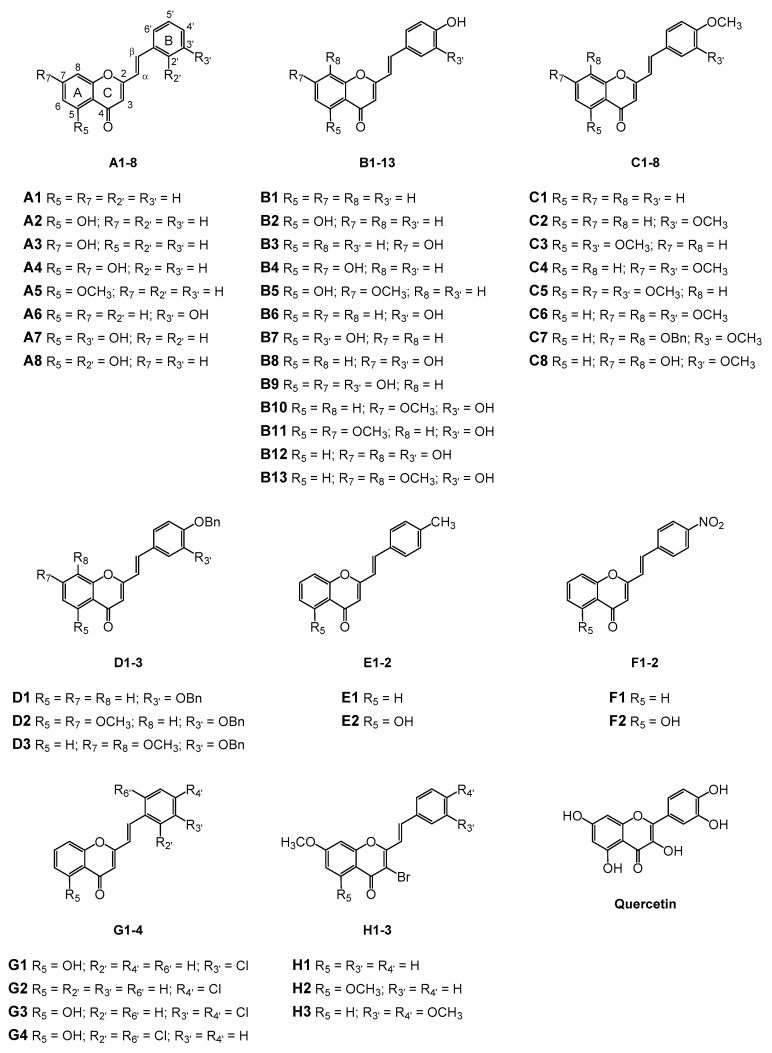
Chemical structures of the studied 2-SC and of the positive control, quercetin. 2-SC were divided into eight groups according to the type of substituents at C-4′ on B-ring (A to F groups), existence and location of -Cl on B-ring (G group), and presence of -Br at C-3 on C-ring (H group).

Styrylchromones are a small group of chromone-derived compounds. Featuring an oxygenated heterocyclic structure, the existence of a styryl moiety attached to the chromone core is one of their characteristic features, and the position of this group determines the nomenclature of these compounds [17]. They are scarcely found in nature and are mostly of synthetic origin. 2-Styrylchromones (2-SC) are the most common type of styrylchromones in nature and their synthesis has been commonly described [17]. Over the years, 2-SC have been reported to display several biological effects, namely antiallergic [18], antiviral [19,20,21], antibacterial, antifungal [22,23,24,25], neuroprotective [26,27,28,29], antioxidant [24,30,31], anti-inflammatory [32,33], and antitumoral [34,35,36,37]. Although the chromone core is present in other type of compounds shown to affect human neutrophils’ viability and oxidative burst [38,39], to the best of our knowledge, such effects have not been demonstrated for 2-SC. The present study intends to fill this gap by evaluating the effect of 43 structurally related 2-SC (Figure 3) on neutrophils’ life span and oxidative burst of freshly isolated human neutrophils, establishing, whenever possible, a structure-activity relationship (SAR).

## 2. Results

### 2.1. Effect of 2-SC on Neutrophils’ Viability

The effects of each 2-SC were evaluated in terms of total neutrophils’ population percentage, and proportion of viable cells [annexin V (−)/PI (−)], apoptotic cells [annexin V (+)/PI (−)], late apoptotic cells [annexin V (+)/PI (+)], and necrotic cells [annexin V (−)/PI (+)].

**Figure 4:**
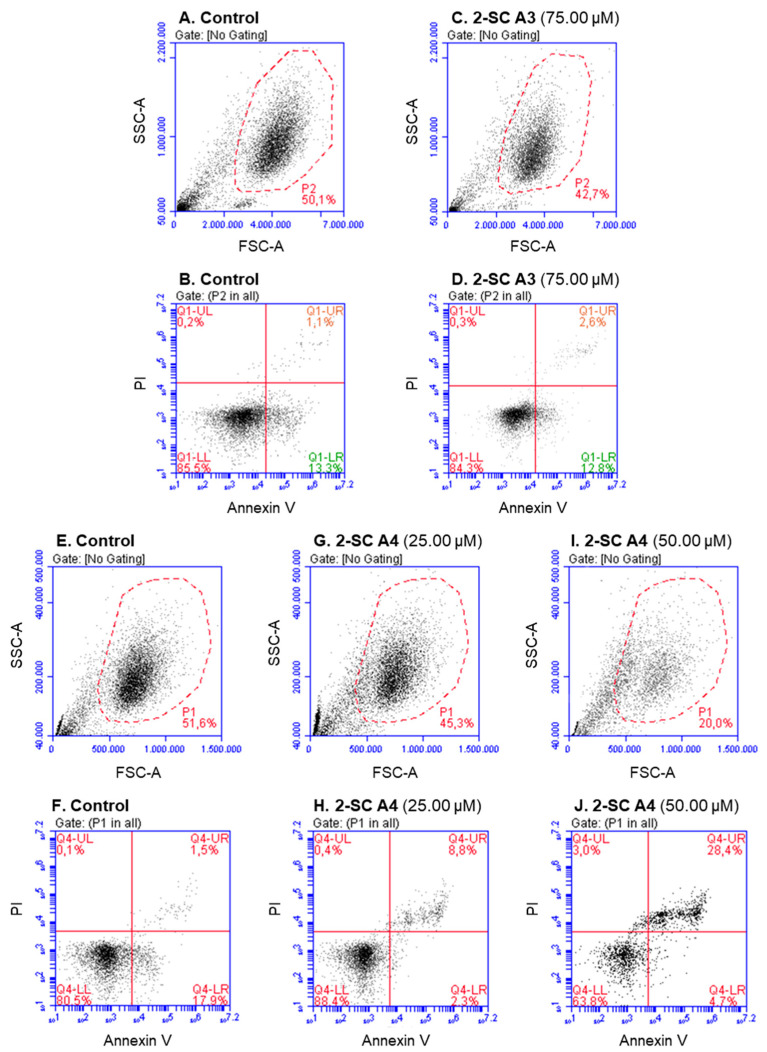
Effect of 2-SC **A3** and **A4** on human neutrophils’ viability. Representative flow cytometry plots of forward scatter area (FSC-A, xx axis) / side scatter area (SSC-A, yy axis) histogram (**A**,**C**,**E**,**G**,**I**), and annexin V (xx axis) / propidium iodide (PI, yy axis) histogram (**B**,**D**,**F**,**H**,**J**). (**A**,**B**,**E**,**F**): control (without 2-SC); (**C**,**D**): 2-SC **A3** (75.00 μM); and (**G**–**J**): 2-SC **A4** (25.00 and 50.00 μM).

At relatively high concentrations, some of the studied 2-SC were cytotoxic. Among the 2-SC of the A group, **A2** (12.50 and 25.00 μM), **A4** (50.00 μM), **A5** (12.50 and 25.00 μM), and **A7** (12.50 and 25.00 μM) significantly affected the total neutrophils’ population percentage and additionally, **A4** and **A5** induced late apoptosis. As an example, Figure 4 displays representative plots of 2-SC **A3** and **A4**, where **A3** (75.00 μM) showed no effect on human neutrophils’ viability, and **A4** (50.00 μM) affected the total neutrophils’ population percentage and induced late apoptosis. Thus, for 2-SC **A4**, it was necessary to decrease the tested concentration to find the one that did not affect neutrophils’ viability (25.00 μM).

However, 2-SC in the B group were the most cytotoxic. 2-SC **B1** (50.00 μM), **B2** (12.50 μM), **B4** (12.50 μM), **B5** (12.50 and 25.00 μM), and **B7** (6.25 μM) affected the total neutrophils’ population percentage, and additionally **B7** (0.75–6.25 μM) induced necrosis. 2-SC **B10** (12.50 μM) was shown to induce late apoptosis. 2-SC **F2** (25.00 μM) induced apoptosis, while **H1** (25.00 μM) affected the total neutrophils’ population percentage and induced late apoptosis. The other studied 2-SC were not cytotoxic at the maximum tested concentration.

**Figure 5:**
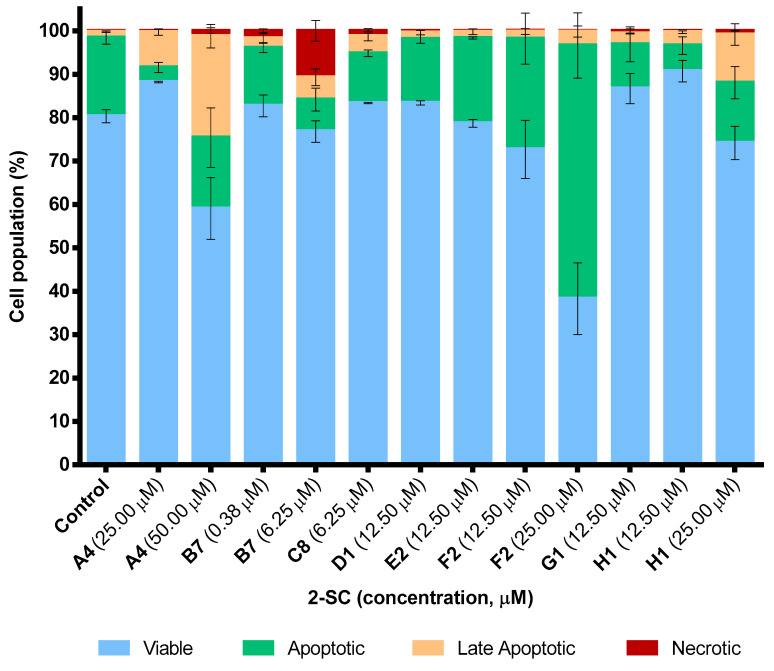
Representative examples of the effect of 2-SC on neutrophils’ viability, for different concentrations: **A4** (25.00 and 50.00 μM), **B7** (0.38 and 6.25 μM), **C8** (6.25 μM), **D1** (12.50 μM), **E2** (12.50 μM), **F2** (12.50 and 25.00 μM), **G1** (12.50 μM), and **H1** (12.50 and 25.00 μM). Results are expressed as the percentages (%) of viable cells [annexin V (−)/PI (−)], apoptotic cells [annexin V (+)/PI (−)], late apoptotic cells [annexin V (+)/PI (+)], and necrotic cells [annexin V (−)/PI (+)]; and presented as mean ± SEM (n ≥ 3).

Figure 5 displays representative examples of the proportion of viable cells, apoptotic cells, late apoptotic cells, and necrotic cells for one 2-SC from each group. 2-SC **F2** (25.00 μM) induced apoptosis when compared to the control, while **A4** (50.00 μM) and **H1** (25.00 μM) induced late apoptosis. The 2-SC **B7** (0.75–6.25 μM) of B group significantly induced necrosis, in a concentration-dependent manner, up to the concentration of 0.38 μM, for which no effect on the viability was observed. 2-SC **C8** (6.25 μM), **D1** (12.50 μM), **E2** (12.50 μM), and **G1** (12.50 μM) did not affect cells’ viability.

The finding of cytotoxic effects allowed us to accurately select the non-toxic concentrations. Table 1 shows the 2-SC concentrations that did not affect human neutrophils’ viability, i.e., the values of viable cells, apoptotic cells, late apoptotic cells, and necrotic cells that were not statistically different from the control (untreated cells). These concentrations were chosen as the maximal concentrations to be tested in the neutrophils’ oxidative burst assay.

### 2.2. Effect of 2-SC on Neutrophils’ Oxidative Burst

**Figure 6:**
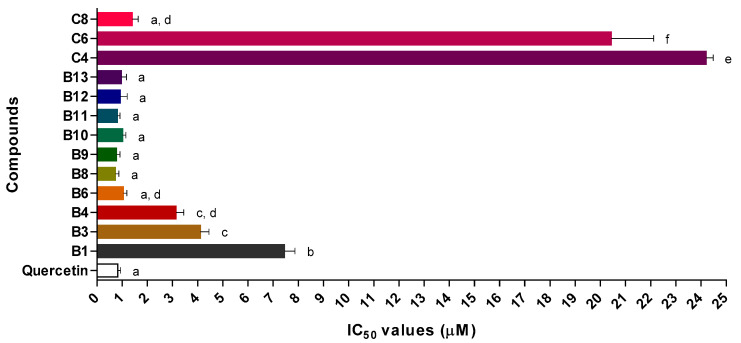
Graphical representation of IC_50_ values of active 2-SC and the positive control, quercetin (μM, mean ± SEM, n ≥ 3). Same letters indicate that IC_50_ values are not statistically different and different letters indicate that IC_50_ values are statistically different from each other (*p* < 0.05).

From all the tested 2-SC, 17 of them were effective inhibitors of neutrophils’ oxidative burst, particularly those belonging to B group, as shown in Table 2. 2-SC **B6**, **B8**–**B13**, and **C8** were the most active compounds, presenting IC_50_ values between 0.7 ± 0.1 (**B8**) and 1.4 ± 0.2 μM (**C8**) (Figure 6). For 2-SC from A group, it was only possible to define the percentage of inhibition of 2-SC **A3**, which reached an inhibitory activity of 53 ± 4% for the concentration of 75.00 μM. The remaining 2-SC in this group were not active, up to the highest tested concentrations (6.25–100.00 μM). Most of the 2-SC from B group inhibited neutrophils’ oxidative burst (IC_50_ values between 0.7 ± 0.1 and 7.5 ± 0.4 μM), except for **B5** and **B7** that did not show an inhibitory effect, but the maximal concentrations able to be tested were 6.25 and 0.38 μM, respectively. 2-SC **B2** only reached 50 ± 3% of inhibition for the concentration of 6.25 μM. Among 2-SC in C group, 2-SC **C8** was the most active, with an IC_50_ value of 1.4 ± 0.2 μM. 2-SC **C4** and **C6** also demonstrated activity (IC_50_ = 24.2 ± 0.3 and 20 ± 2 μM, respectively), whereas **C3** and **C5** only reached 54 ± 4% and 54 ± 2% of inhibition, respectively, for the concentration of 50.00 μM. 2-SC from D–H groups did not show an effect on the neutrophils’ oxidative burst, up to the maximum tested concentrations (6.25–50.00 μM).

## 3. Discussion

During the inflammatory response, there is an increase in the production of reactive species by neutrophils, and consequently, an increase in oxygen consumption, leading to an overproduction of reactive oxygen species (ROS) and reactive nitrogen species (RNS)—oxidative burst [7,40]. The modulation of the oxidative burst can occur by scavenging of reactive species and/or inhibition of the enzymes directly involved in the production of reactive species, namely NADPH oxidase complex, superoxide dismutase, and MPO [9].

2-SC have demonstrated various biological activities, including antioxidant and anti-inflammatory [17]. However, to the best of our knowledge, there are no studies in the literature about the effects of 2-SC on human neutrophils’ viability and oxidative burst.

The studied panel of structurally related 2-SC was divided into eight groups considering the presence and the type of substituents (none, -OH, -OCH_3_, -OBn, -CH_3_, and -NO_2_) at C-4′ on B-ring (A to F groups), presence and location of -Cl on B-ring (G group), and presence of -Br at C-3 on C-ring (H group), which allowed to establish a SAR. As mentioned, this study is divided into two parts: the evaluation of the effects of 2-SC on neutrophils’ viability and modulation of the oxidative burst.

In the first part of this work, annexin V/PI flow cytometric assay was applied to assess human neutrophils’ viability. In what concerns neutrophils’ viability, the majority of the studied 2-SC did not affect the viability of these cells, up to the maximum concentration tested for each compound. However, some 2-SC were cytotoxic, affecting the total neutrophils’ population percentage (**A2**, **A4**, **A5**, **A7**, **B1**, **B2**, **B4**, **B5**, **B7**, and **H1**) and inducing apoptosis (**F2**), late apoptosis (**A4**, **A5**, **B10**, and **H1**), or directly necrosis (**B7**). Currently, in the literature, some of the 2-SC studied in this work are described as being cytotoxic at lower concentrations; however, to the best of our knowledge, there are only studies demonstrating this cytotoxicity in tumor cell lines [34,35,41,42]. In the present work, the concentrations at which the 2-SC showed cytotoxicitywill not have an impact at a physiological level, since they are relatively high (>6.25 μM). Although there are no studies regarding the metabolism and bioavailability of 2-SC, they can be compared with flavonoids, since they are structurally related compounds to 2-SC. It is described in the literature the concentration of flavonoids that can be found in human plasma rarely exceeds 1 μM [43].

In the next phase of this work, a chemiluminescence method, using luminol as a probe, was applied to evaluate the modulation of neutrophils’ oxidative burst. Among the studied 2-SC, the ones from B group were the most effective. Regarding 2-SC from A group, no significant effect in the modulation of the oxidative burst was found. The 2-SC of this group have no substituent at C-4′ on B-ring. Some of the 2-SC belonging to A group (**A1**–**A4**) were evaluated by Gomes and co-workers [30] for their scavenging reactive species potential, but were not shown very effective. Ribeiro and co-workers [38] used the same methodology of the present work to assess the modulatory effect of oxidative burst of some flavonoids structurally similar to these 2-SC (**A1**–**A4**, **A6**, and **A7**); however, there was no significant difference between the activity of flavonoids and 2-SC, since both showed low or no activity.

As mentioned, most of the 2-SC with the best activity belong to B group. These 2-SC have in common the presence of -OH group at C-4′ on B-ring. This substituent in this position in the 2-SC structure (**B1**) seems to contribute to the activity, when compared with the unsubstituted **A1**. Nevertheless, it should be noted that within this group, the presence of a catechol group at C-3′ and C-4′ on B-ring seems to be more favorable for the intended activity, as **B6** was almost 7.5 times more active than **B1**. Gomes and co-workers [30,44] showed that some of the 2-SC from this group (**B1**, **B3**, **B4**, **B6**–**B9**, **B12**, and **B13**) were effective scavengers of reactive species, namely superoxide anion radical (O_2_^•^^−^), hydrogen peroxide (H_2_O_2_), hypochlorous acid (HOCl), singlet oxygen (^1^O_2_), nitric oxide radical (^•^NO), and peroxynitrite anion (ONOO^−^), while **B2** was much less active or inactive. Interestingly, the 2-SC that were the most effective in scavenging reactive species (**B6**, **B8**, **B9**, **B12**, and **B13**) were also good modulators of the oxidative burst. This suggests that these 2-SC may modulate oxidative burst by scavenging reactive species. Ribeiro and co-workers [38] also evaluated some flavonoids structurally identical to some of the studied 2-SC (**B1**–**B4** and **B6**–**B9**), differing only in the presence of the styryl moiety. Overall, the 2-SC showed a better effect than the correspondent flavonoid, which may indicate that the styryl moiety contributes to the observed enhanced effect.

The structures of the 2-SC of B group differ from those of A group mainly by the presence of -OH groups on B-ring. The presence of -OH groups on B-ring of the 2-SC has a greater influence on inhibitory activity than the presence of -OH on A-ring, as can be seen by comparing the activities found for **B6** and **A4**. **B6** was active while **A4** showed no activity. This observation was also corroborated when -OCH_3_ groups are present on the A-ring, as can be seen by comparing **B8** with **B10**, **B9** with **B11**, and **B12** with **B13**. These results seem to indicate that the effects of 2-SC are mainly influenced by the presence of -OH group on B-ring, namely the presence of the catechol at C-3′ and C-4′. The importance of the catechol group for the antioxidant activity seems to be related to the formation of a semiquinone, which suffers dismutation and forms a stable ortho-quinone. This process is possible since the -OH groups are highly reactive and able to donate an electron to the reactive species, which stabilizes them due to electron delocalization [45,46].

Among the 2-SC in C group, 2-SC **C8** was the most active. **C8** has a catechol group at C-7 and C-8 on A-ring, whereas on B-ring it has -OCH_3_ groups at C-3′ and C-4′. Previously, **C8** also demonstrated to have a good scavenging activity of reactive species (O_2_^•^^−^, ^1^O_2_, ^•^NO and ONOO^−^) [44]. **C4** and **C6** also showed some activity but were about 14 and 17 times less active, respectively, than **C8**. In fact, **C4** and **C6** have no -OH group in their structure, unlike **C8**, demonstrating the importance of -OH groups for this effect. This idea can also be corroborated by comparing the structure of **C1** with **B1**, both with only one substituent present at C-4′ on B-ring: **B1** has -OH and **C1** has -OCH_3_, and, as expected, **B1** was active while **C1** was not active. The same behavior was observed between **B6** and **C2**, where **B6** presents a catechol at C-3′ and C-4′ on B-ring, and **C2** presents -OCH_3_ groups at the same positions. When two -OCH_3_ groups are present on B-ring, namely at C-3′ and C-4′, the presence of one -OCH_3_ at C-7 on A-ring (**C4**) seems to better improve the activity when compared to one -OCH_3_ at C-5 on A-ring (**C3**). When only -OCH_3_ groups are present as substituents in 2-SC, the presence of two -OCH_3_ on A-ring only enhanced the activity if they are on C-7 and C-8, as can be seen by comparing **C6** with **C5**. **C6** has -OCH_3_ at C-7 and C-8 on A-ring and C-3′ and C-4′ on B-ring, while on **C5** the -OCH_3_ groups are located at C-5 and C-7 on A-ring and C-3′ and C-4′ on B-ring. Still, in the presence of two -OCH_3_ groups on B-ring, varying the type of substituents present on A-ring, namely at C-7 and C-8, it can be concluded that -OH group (**C8**) is the one that most favors the modulation of the oxidative burst, when compared to the presence of -OCH_3_ (**C6**) and -OBn (**C7**). The presence of -OBn groups on B-ring also did not favor the activity as observed in the results obtained for 2-SC **D1**–**D3**. The same behavior was also observed for the 2-SC with -CH_3_ (**E1** and **E2**) or -NO_2_ (**F1** and **F2**) on C-4′ on B-ring. The presence of -Cl groups at different positions on B-ring also does not contribute to the activity of **G1**–**G4**, even in the presence of -OH groups on C-5 on A-ring (**G1**, **G3**, and **G4**). The simultaneous existence of -Br at C-3 on C-ring and -OCH_3_ at C-7 on A-ring (**H1**–**H3**) also did not seem to favor the modulation of oxidative burst.

**Figure 7:**
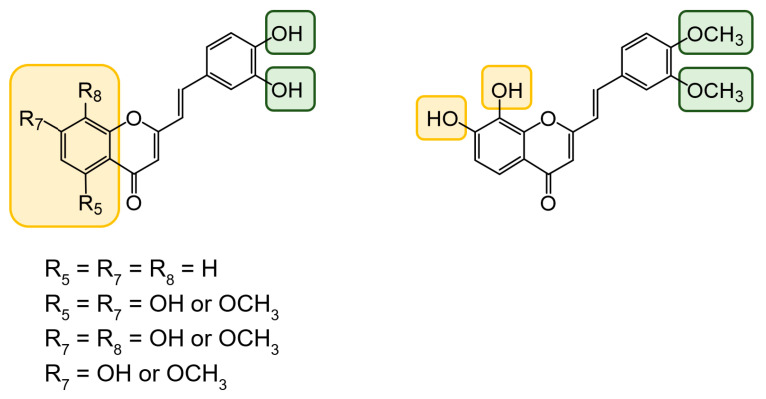
Chemical structures of the most active 2-SC in the modulation of neutrophils’ oxidative burst.

Figure 7 shows the chemical structures and structural characteristics of the most active 2-SC in the modulation of human neutrophils’ oxidative burst.

## 4. Materials and Methods

### 4.1. Reagents

Histopaque 1077 and 1119, trypan blue solution 0.4%, calcium chloride (CaCl_2_), Trizma^®^ base, D-glucose, quercetin, magnesium sulfate (MgSO_4_), Dulbecco’s phosphate buffer saline, without CaCl_2_ and magnesium chloride (PBS), phorbol-12-myristate-13-acetate (PMA), dimethylsulfoxide (DMSO), and luminol were obtained from Sigma Chemical Co. (St. Louis, MO, USA). Sodium chloride (NaCl) and potassium chloride (KCl) were obtained from VWR Chemicals (Alfragide, Portugal) and Pronalab (Abrunheira, Portugal), respectively. The commercial FITC Annexin V Apoptosis Detection Kit I was obtained from BD Biosciences (Franklin Lakes, NJ, USA). All compounds were synthesized as previously described [44,47,48].

### 4.2. Equipment

The determination of cells’ yield and viability, through the trypan blue exclusion method, was done on an optic microscope (Nikon Eclipse E200, Nikon Instruments Inc., Melville, NY, USA). The Accuri C6 flow cytometer (BD, Becton, Dickinson and Company, Franklin Lakes, NJ, USA) was used to collect the fluorescence signals for the assessment of 2-SC effects on neutrophils’ viability (apoptosis and necrosis). The chemiluminescent assay for the in the assessment of 2-SC effects on neutrophils’ oxidative burst was performed in a microplate reader (Synergy HT, BIO-TEK Instruments, Inc., Winooski, VT, USA).

### 4.3. Methods

#### 4.3.1. General Information

All the studied 2-SC and the positive control, quercetin, were dissolved in DMSO (4% in the reactional mixture). Preliminary experiments were performed to verify the clear solubility of the 2-SC under study in the tested experimental conditions and their possible interference effects with the used methodologies. From these assays, the maximum tested concentration was chosen, to avoid any interference with the methodologies used. At least three individual independent experiments were performed in duplicate, for each assay, using between three and nine concentrations.

#### 4.3.2. Human Neutrophils Isolation

Following the Declaration of Helsinki and the approval of patient-related procedures and protocols by the Ethics Committee of Centro Hospitalar do Porto, Portugal, venous blood was collected from healthy human blood donors, after informed consent. The blood was stored into K_3_EDTA vacuum tubes after antecubital venipuncture of the donors. In order to obtain the isolated neutrophils, the density gradient centrifugation method was applied, as previously reported [38], with modifications. In brief, in a 15 mL polypropylene tube, 3 mL of histopaque 1119 were added followed by 3 mL of histopaque 1077 and finally, 4.5 mL of the collected blood were slowly added. Each tube was centrifuged at 900× *g* at 20 °C for 30 min, with a medium acceleration and deceleration speed. Following centrifugation, the neutrophils’ portion was harvested, and PBS was added to double the volume. The neutrophils’ suspension was then centrifuged at 850× *g* at 4 °C for 5 min, using a maximum acceleration and deceleration speed. The supernatant was discarded, and the neutrophils’ pellet was resuspended in PBS and afterward milli-Q water was added to lyse the residual erythrocytes. The tube was gently homogenized and after a few minutes, 3% NaCl was added to restore isotony, and then centrifuged again at 850× *g* at 4 °C for 5 min, using a maximum acceleration and deceleration speed. The supernatant was discarded, and a neutrophils’ pellet was obtained. After isolation, the neutrophils’ pellet was resuspended in the incubation media, tris-glucose buffer (25 mM Trizma^®^ base, 5.5 mM D-glucose, 140 mM NaCl, 1.26 mM CaCl_2_, 0.81 mM MgSO_4_, 5.37 mM KCl, pH = 7.4). Neutrophils’ suspension was kept on ice, under soft shaking, until use. The trypan blue exclusion method was used to determine cells’ yield and viability.

#### 4.3.3. Assessment of the Effect of 2-SC on Neutrophils’ Viability

The effect of the studied 2-SC on neutrophils’ viability was assessed by flow cytometry, after simultaneous staining with FITC Annexin V and propidium iodide (PI) (Figure S1), as previously reported by our research group [49], with modifications. After isolation, neutrophils (1 × 10^6^ cells/mL) were incubated with 2-SC (up to 100.00 μM) or DMSO for 40 min, at 37 °C, in a 48-well plate. At the end of incubation, the content of each well was transferred to conical microtubes and centrifuged at 400× *g* at 20 °C for 5 min. After discarding the supernatant, the neutrophils’ pellet was resuspended in PBS and centrifuged under the previous conditions. Once the supernatant was removed, the pellet was resuspended in 10× diluted binding buffer, and then PI and annexin V were added. This mixture was incubated in the dark at room temperature for 15 min, and finally, 10× diluted binding buffer was added. In a flow cytometer, the fluorescence signal, of at least 10,000 cells/sample, were collected in logarithmic mode and followed in channels 1 and 3. The green fluorescence corresponding to annexin V FITC was accompanied in channel 1 and plotted as a histogram of FL1 staining, whereas the fluorescence corresponding to PI was monitored in channel 3 and plotted in the same histogram but as FL3 staining. The collected data were analyzed with the BD Accuri™ C6 software. The data analysis was restricted to the neutrophils’ population. Thus, according to the light-scattering properties of neutrophils (in a forward versus side scatter plot), a polygon gate was drawn, where debris and other blood cells were excluded. The effect of 2-SC on neutrophils’ viability was expressed as a relative percentage of apoptotic and/or necrotic cells when compared to the control (without 2-SC).

#### 4.3.4. Assessment of the Effect of 2-SC on Neutrophils’ Oxidative Burst

The effect of 2-SC on the modulation of neutrophils’ oxidative burst was assessed after the stimulation of these cells with PMA by monitoring the oxidation of chemiluminescent probe, luminol, by the generated reactive species (Figure S2), according to a previously described methodology [38]. Briefly, neutrophils (1 × 10^6^ cells/mL) were pre-incubated with luminol (500.00 μM), and 2-SC (0.00–100.00 μM) or DMSO, at the final concentrations indicated. After this pre-incubation, at 37 °C for 5 min, PMA (160.00 nM) was added, and in turn, neutrophils’ stimulation was initiated. Kinetic readings were immediately initiated and followed for 40 min, at 37 °C, in a microplate reader. The arbitrary chemiluminescence value was taken at the peak of the curve, and the results were expressed as the inhibition percentage of luminol oxidation. The maximum tested concentrations of 2-SC in this assay were defined according to the results obtained in the viability assay, where the concentrations that did not affect neutrophils’ viability were defined.

#### 4.3.5. Statistical Analysis

GraphPad Prism 6 software was used to plot the curves of percentage of inhibition versus concentrations of compound, from which the concentration that produces 50% of inhibition (IC_50_) was determined. GraphPad Prism 6 software was also used to perform all statistical analyses. Statistical comparison among the most active 2-SC was estimated by applying the one-way analysis (ANOVA), followed by the Bonferroni’s multiple comparisons test. In all cases, *p*-values < 0.05 were considered statistically significant. The results are expressed as mean ± standard error of the mean (SEM).

## 5. Conclusions

In conclusion, the effect of 2-SC on human neutrophils’ viability and oxidative burst was assessed for the first time in this study, providing promising results. Most of the studied 2-SC did not affect neutrophils’ viability, at physiologically meaningful concentrations. The 2-SC belonging to B group, with only one -OH group at C-4′ or a catechol group at C-3′ and C-4′ on B-ring, were the most effective, which seems to indicate that the presence and the position of these groups play an important role for the modulation of human neutrophil’s oxidative burst. Additionally, the 2-SC with -OCH_3_ groups at C-3′ and C-4′ on B-ring (C group) also showed modulatory effects, nonetheless not as effective, with exception of **C8**. One of the possible mechanisms of action of the most active 2-SC found may be through the scavenging of reactive species, since they have already demonstrated this ability in in vitro non-cellular studies.

The obtained results in this study demonstrate that 2-SC are safe to human neutrophils and simultaneously display a scaffold with great potential for the development of new drugs to act on inflammatory processes, especially when a catechol group is present at the B-ring.

## Figures and Tables

**Table 1:** Higher 2-SC concentrations (μM) that did not affect human neutrophils’ viability, according to the annexin V/PI assay.

2-SC	Concentration (μM)	2-SC	Concentration (μM)
**A1**	75.00	**C2**	25.00
**A2**	6.25	**C3**	50.00
**A3**	75.00	**C4**	37.50
**A4**	25.00	**C5**	75.00
**A5**	6.25	**C6**	37.50
**A6**	100.00	**C7**	25.00
**A7**	6.25	**C8**	6.25
**A8**	25.00	**D1**	12.50
**B1**	25.00	**D2**	12.50
**B2**	6.25	**D3**	12.50
**B3**	25.00	**E1**	25.00
**B4**	6.25	**E2**	12.50
**B5**	6.25	**F1**	6.25
**B6**	12.50	**F2**	12.50
**B7**	0.38	**G1**	12.50
**B8**	25.00	**G2**	12.50
**B9**	12.50	**G3**	25.00
**B10**	6.25	**G4**	25.00
**B11**	12.50	**H1**	12.50
**B12**	12.50	**H2**	50.00
**B13**	12.50	**H3**	12.50
**C1**	25.00		

**Table 2:** Chemical structures and inhibition of human neutrophils’ oxidative burst by the tested 2-SC and the positive control, quercetin.

2-SC		R_5_	R_7_	R_8_	R_2′_	R_3′_	R_4′_	R_6′_	Inhibitory Activity * (% ± SEM) or IC_50_ (μM, mean ± SEM)
**A1**	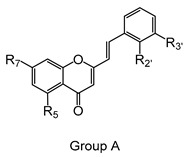	-	-	-	-	-	-	-	<30% ^75.00µM^
**A2**		OH	-	-	-	-	-	-	<30% ^6.25µM^
**A3**		-	OH	-	-	-	-	-	53 ± 4 % ^75.00µM^
**A4**		OH	OH	-	-	-	-	-	<30% ^25.00µM^
**A5**		OCH_3_	-	-	-	-	-	-	<30% ^6.25µM^
**A6**		-	-	-	-	OH	-	-	<30% ^100.00µM^
**A7**		OH	-	-	-	OH	-	-	<30% ^6.25µM^
**A8**		OH	-	-	OH	-	-	-	<30% ^25.00µM^
**B1**	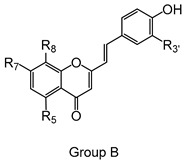	-	-	-	-	-	-	-	7.5 ± 0.4
**B2**		OH	-	-	-	-	-	-	50 ± 3 % ^6.25µM^
**B3**		-	OH	-	-	-	-	-	4.1 ± 0.3
**B4**		OH	OH	-	-	-	-	-	3.1 ± 0.3
**B5**		OH	OCH_3_	-	-	-	-	-	<30% ^6.25µM^
**B6**		-	-	-	-	OH	-	-	1.0 ± 0.1
**B7**		OH	-	-	-	OH	-	-	<30% ^0.38µM^
**B8**		-	OH	-	-	OH	-	-	0.7 ± 0.1
**B9**		OH	OH	-	-	OH	-	-	0.8 ± 0.1
**B10**		-	OCH_3_	-	-	OH	-	-	1.0 ± 0.1
**B11**		OCH_3_	OCH_3_	-	-	OH	-	-	0.8 ± 0.1
**B12**		-	OH	OH	-	OH	-	-	0.9 ± 0.3
**B13**		-	OCH_3_	OCH_3_	-	OH	-	-	1.0 ± 0.2
**C1**	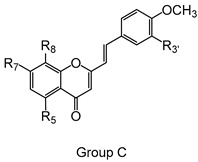	-	-	-	-	-	-	-	<30% ^25.00µM^
**C2**		-	-	-	-	OCH_3_	-	-	<30% ^25.00µM^
**C3**		OCH_3_	-	-	-	OCH_3_	-	-	54 ± 4 % ^50.00µM^
**C4**		-	OCH_3_	-	-	OCH_3_	-	-	24.2 ± 0.3
**C5**		OCH_3_	OCH_3_	-	-	OCH_3_	-	-	54 ± 2 % ^50.00µM^
**C6**		-	OCH_3_	OCH_3_	-	OCH_3_	-	-	20 ± 2
**C7**		-	OBn	OBn	-	OCH_3_	-	-	<30% ^25.00µM^
**C8**		-	OH	OH	-	OCH_3_	-	-	1.4 ± 0.2
**D1**	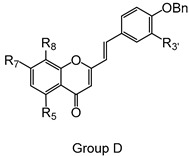	-	-	-	-	OBn	-	-	<30% ^12.50µM^
**D2**		OCH_3_	OCH_3_	-	-	OBn	-	-	<30% ^12.50µM^
**D3**		-	OCH_3_	OCH_3_	-	OBn	-	-	<30% ^12.50µM^
**E1**	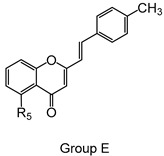	-	-	-	-	-	-	-	<30% ^6.25µM^
**E2**		OH	-	-	-	-	-	-	<30% ^12.50µM^
**F1**	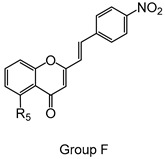	-	-	-	-	-	-	-	<30% ^6.25µM^
**F2**		OH	-	-	-	-	-	-	<30% ^12.50µM^
**G1**	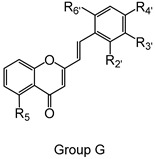	OH	-	-	-	Cl	-	-	<30% ^12.50µM^
**G2**		-	-	-	-	-	Cl	-	<30% ^12.50µM^
**G3**		OH	-	-	-	Cl	Cl	-	<30% ^25.00µM^
**G4**		OH	-	-	Cl	-	-	Cl	<30% ^25.00µM^
**H1**	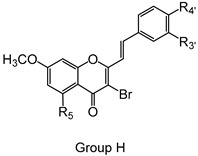	-	-	-	-	-	-	-	<30% ^12.50µM^
**H2**		OCH_3_	-	-	-	-	-	-	<30% ^50.00µM^
**H3**		-	-	-	-	OCH_3_	OCH_3_	-	<30% ^12.50µM^
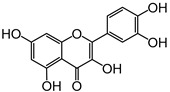		Positive control Quercetin							0.8 ± 0.1

* The percentage of inhibition is expressed for the highest concentration (in superscript) that could be tested under the assay conditions to avoid interferences with the methodology.

## Data Availability

Data are contained within the article.

## Supplementary Materials

The following supporting information can be downloaded at: www.mdpi.com/article/10.3390/ph15030288/s1, Figure S1: Annexin V/propidium iodide (PI) staining, Figure S2: Luminol staining.
