# Supplementary material for: 2-Styrylchromones: Cytotoxicity and Modulation of Human Neutrophils’ Oxidative Burst
Source: Pharmaceuticals (Basel). 2022 Feb 25;15(3):288. doi: 10.3390/ph15030288 (PMC8953385; doi:10.3390/ph15030288)
Supplement: Supplementary file 1 [file pharmaceuticals-15-00288-s001.zip › pharmaceuticals-1596034-supplementary.pdf]

2-Styrylchromones: Cytotoxicity and Modulation of Human Neutrophils' Oxidative Burst

Mariana Lucas [1], Marisa Freitas [1], Marco Zanchetta [2], Artur M.S. Silva [2], Eduarda Fernandes [1*], Daniela Ribeiro [1,3*]

[a] LAQV, REQUIMTE, Laboratory of Applied Chemistry, Department of Chemical Sciences, Faculty of Pharmacy, University of Porto, 4050-313 Porto, Portugal; mflucas@ff.up.pt (M.L.); marisafreitas@ff.up.pt (M.F.)
[b] LAQV, REQUIMTE & Department of Chemistry, University of Aveiro, Campus de Santiago, 3810-193 Aveiro, Portugal; m.zanchetta3@campus.unimib.it (M.Z.); artur.silva@ua.pt (A.M.S.S.)
[c] Faculty of Agrarian Sciences and Environment, University of the Azores, 9700-042 Angra do Heroísmo, Açores, Portugal.
* Correspondence: egracas@ff.up.pt (E.F.); daniela.sa.ribeiro@uac.pt (D.R.)

Supplementary Material

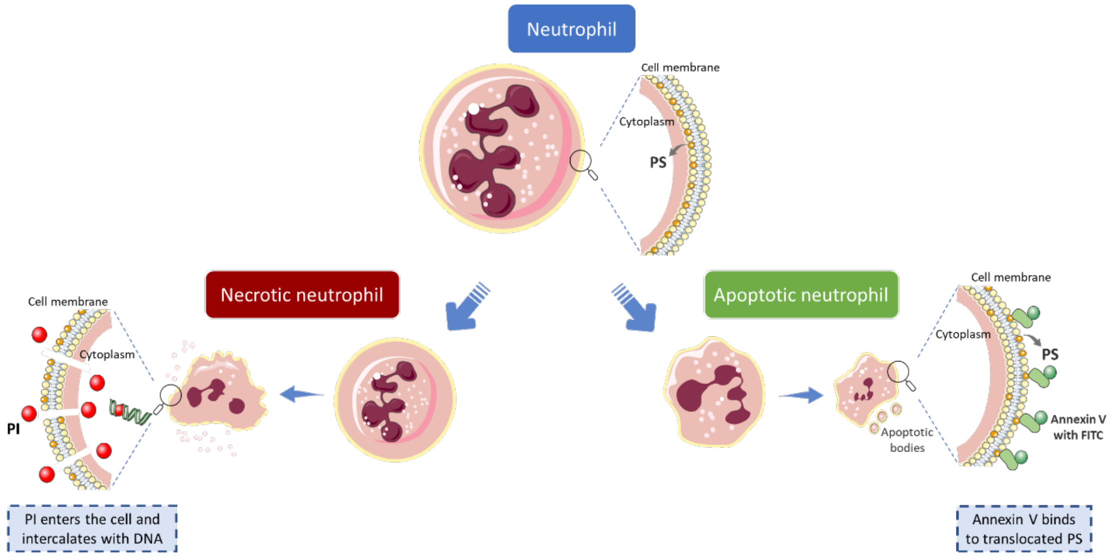

Figure S1. Annexin V / propidium iodide (PI) staining. During necrosis and after PI staining, this probe enters the necrotic cells whose membrane is already disintegrated and intercalates into the grooves of DNA, producing a red fluorescent adduct that can be detected by flow cytometry. In the apoptotic process changes in cell structure occur, phosphatidylserine (PS) translocates from the inner side of the membrane to the outer side, which allows annexin V [conjugated with a fluorescent label, fluorescein isothiocyanate (FITC)] to bind to these residues, and to detect apoptosis [13,15,16].

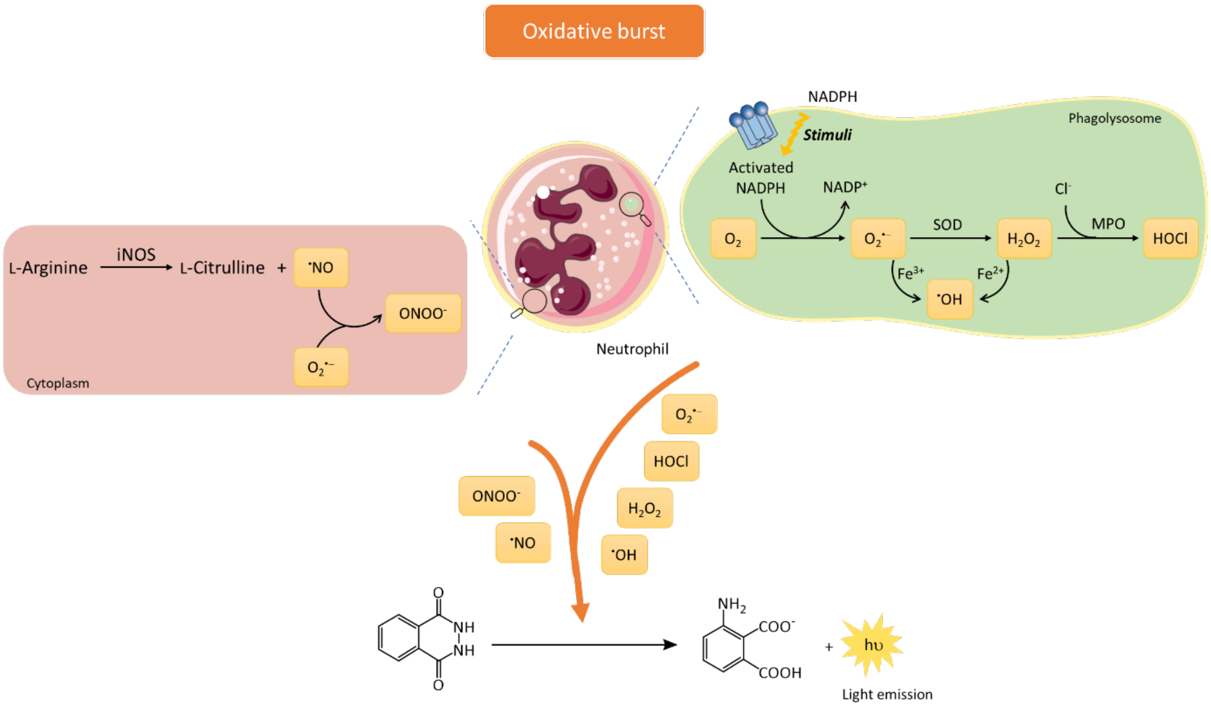

Figure S2. Luminol staining. This probe can diffuse across the cell membrane and detect extra- and intracellularly produced reactive species. In the presence of reactive species [superoxide anion radical ($O_2^{\bullet-}$), hydrogen peroxide (H_2O_2), hypochlorous acid (HOCl), hydroxyl radical (•OH), nitric oxide radical (•NO), peroxynitrite anion ($ONOO^-$)], luminol reacts with them, originating an energetically excited molecule, the aminophtalate, that when returning to the ground state emits light [9].
